# Design and Fabrication of Capillary-Driven Flow Device for Point-Of-Care Diagnostics

**DOI:** 10.3390/bios10040039

**Published:** 2020-04-15

**Authors:** Sammer-ul Hassan, Xunli Zhang

**Affiliations:** 1 Bioengineering Research Group, Faculty of Engineering and Physical Sciences, University of Southampton, Southampton SO17 1BJ, UK; 2 Institute for Life Sciences, University of Southampton, Southampton SO17 1BJ, UK

**Keywords:** microfluidics, point-of-care (POC) diagnostics, β-lactamase, lab-on-a-chip, capillary-driven flow, colorimetry, optical detections, smartphone imaging, analytical chemistry

## Abstract

Point-of-care (POC) diagnostics enables the diagnosis and monitoring of patients from the clinic or their home. Ideally, POC devices should be compact, portable and operatable without the requirement of expertise or complex fluid mechanical controls. This paper showcases a chip-and-dip device, which works on the principle of capillary-driven flow microfluidics and allows analytes’ detection by multiple microchannels in a single microchip via smartphone imaging. The chip-and-dip device, fabricated with inexpensive materials, works by simply dipping the reagents-coated microchip consisting of microchannels into a fluidic sample. The sample is loaded into the microchannels via capillary action and reacts with the reagents to produce a colourimetric signal. Unlike dipstick tests, this device allows the loading of bacterial/pathogenic samples for antimicrobial testing. A single device can be coated with multiple reagents, and more analytes can be detected in one sample. This platform could be used for a wide variety of assays. Here, we show the design, fabrication and working principle of the chip-and-dip flow device along with a specific application consisting in the determination of β-lactamase activity and cortisol. The simplicity, robustness and multiplexing capability of the chip-and-dip device will allow it to be used for POC diagnostics.

## 1. Introduction

Capillary-driven flow microfluidics is a type of microfluidics which allows fluid movements into microchannels via capillary forces and does not require internal/external expensive and complicated fluid management mechanisms [1]. This type of microfluidics has the potential to provide rapid, inexpensive and simple clinical assays in short times and near the patient. The reagents can be pre-coated on to the surface of the channels, and results are obtained via a portable detector/smartphone by merely dipping the device into the patient sample, after a short incubation. Therefore, this type of system can lead to the development of the ever-promised simple point-of-care (POC) devices sought after by the scientific community for decades [2,3].

Several capillary-driven flow devices have been developed and tested for POC diagnostics applications, such as that of Ramalingam et al. [4], who developed a numerical model to study capillary-driven flow in capillaries for polymerase chain reaction (PCR). The authors numerically modelled and tested polydimethylsiloxane (PDMS) microchips and validated the flow inside the capillaries by tracking the fluid meniscus. Capillary-driven flow devices were also fabricated [5,6] to measure the viscosity of a fluid based on capillary action in microchannels. For example, Lee et al. [5] developed a capillary-driven flow microfluidics device to measure zebrafish blood viscosity in microchannels. The method allowed the validation of the Newtonian fluid behavior and dynamic viscosity of blood, requiring a minute amount of blood from zebrafish.

In another development, PMMA was also used to develop a capillary flow device for nucleic acid biosensing applications using 500 µm-wide microfluidic channels consisting of sealed reagent-loaded pads [7]. Furthermore, capillary-driven flow microfluidics has also been developed for the measurement of biomedically relevant biomarkers [8,9,10,11,12,13]. The dynamics of open microfluidic channels has also been studied via 3D printing of microchannels for rapid prototyping and mass fabrication options [14]. Additionally, capillary flow has also been used for blood plasma separation in microfluidic channels, as reported by Madadi et al. [13], who developed a capillary flow device to separate plasma using 5 µL of sample to obtain 0.1 µL of plasma for diagnostics applications. Delamarche’s group [9,15,16] has widely developed plasma separation devices combined with immunodiagnostics devices, such as a system to detect C-reactive protein (CRP), which was quantified by using 5 µL of human serum extracted from a blood sample and 3.6 nL of a reagent solution deposited on the chip [15]. This type of device has been further developed for multiple biomedical applications such as portable bead-based and immunodiagnostics assays, with the possibility of detection via smartphones or handheld devices [16].

Glass/hydrophilic capillaries have also been used to drive flow via capillary action, as described by Lapierre et al. [17], who used bare glass capillaries to collect blood samples. In contrast, fluoropolymer microcapillaries (FEP) have been coated with reagents to render their surface hydrophilic and draw up blood or aqueous samples in a minute fraction of time [18,19,20,21,22]. Pivetal et al. [19] coated FEP capillaries with polyvinyl alcohol (PVA) to convert their surface from hydrophobic to hydrophilic and attached reagents or antibodies on the surface for the detection of protein biomarkers. The reagents reacted with the biomarkers, generating a colour or fluorescent signals which were detected under a microscope attached to a camera. Similarly, FEP microcapillaries were used to assess prostate-specific antigen (PSA) by an enzymatic reaction [20,21] and cytokines [22] within microcapillaries. The FEP microcapillaries were multiplexed in parallel by injecting solutions into 10 capillaries at the same time, i.e., a PSA standard solution, detection antibodies, the enzyme complex, washing solutions and the enzymatic substrates, which were injected in the capillaries simultaneously. The FEP microcapillaries were placed vertically in a blood sample to draw up liquid for ABO blood typing [18]. As the liquid rose into the capillaries, the reagents were released into the sample and reacted with biomarkers to produce a colour or a fluorescent signal, which was then detected by microscope or portable/smartphone systems.

Capillary-driven flow microfluidics have a great potential as POC diagnostics, for instance, for the prevention of antimicrobial resistance in healthcare [2,18]. Here, we developed a capillary-driven flow device which is simple to operate and allows loading multiple samples in a single device. In this study, the design, fabrication and working principle of the capillary-driven flow device are illustrated along with applications consisting in β-lactamase and cortisol activity assays.

## 2. Experimental

**Figure 1:**
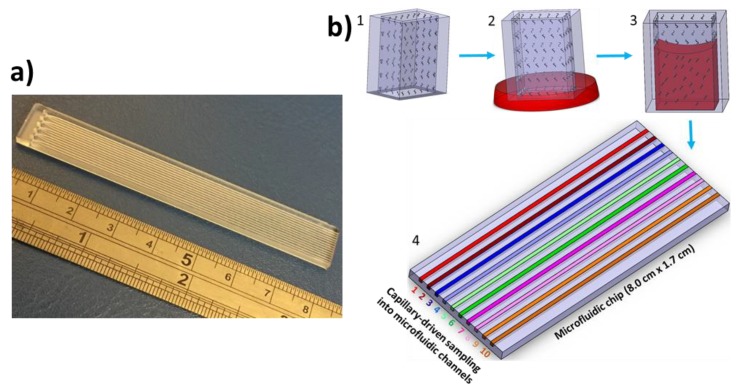
Chip-and-dip device design and working principle. (**a**) Photograph of the chip-and-dip device. (**b**) Schematic of the working principle of the chip-and-dip platform for point-of-care diagnostics in healthcare.

The microchip was designed using the CAD software (SolidWorks, Dassault Systemes), and 8 cm-long microchannels were micromachined on a PMMA piece (1.2 mm of thickness) with dimensions of 0.25 × 0.20 mm (width × depth). Another PMMA piece with similar dimensions was cut for sealing the microchannels. Both pieces were sonicated in a water bath to completely remove the debris from the microchannels or surface and were cleaned with isopropyl alcohol (IPA). The microchannels were air-dried and placed on a glass Petri dish, with the microchannels facing upward. a mixture of ethanol and acetone (50:50) was poured on the PMMA pieces and left for 30 s. The microchip was fabricated by combining the two PMMA pieces for 30 s (the solvent-treated surfaces of both PMMA pieces were bound together producing microchannels), air-drying the microchannels to completely remove the excess of mixture left in the microchannels and pressing with a metal piece (3–4 kg weight) for 5 min. Figure 1 shows a photograph of the fabricated microchip.

The microchip was then placed in an oxygen plasma cleaner (Diener electronics) for 8 min at a power of 90 watts. Then, 2% PVA (146000–186000) was prepared in deionized water (DI) water and used to fill the microchannels at room temperature. The solution was removed from the microchannels after 10 min, and the microchannels were dried by compressed air and left in an oven for 15 min at 60–65 °C. A second solution consisting of 5% PVA, 5 mM glutaraldehyde and 5 mM hydrochloric acid was freshly prepared and injected into the microchannels at room temperature. After 10 min, the solution was removed via compressed air, and the microchip was left for 30 min at room temperature. The microchip coated with PVA was directly used for food dye calibrations. For reagents cross-linking, the microchannels were filled with reagent solutions (3,3′,5,5′-tetramethylbenzidine (TMB) or nitrocefin) at room temperature and left for 30 min. The reagents were then removed from the microchannels via compressed air and used straightaway for the experiments. Figure 1b shows the schematic of the working principle of the chip-and-dip device. Firstly, the device is fabricated with microchannels and coated with reagents as discussed above. Secondly, the device is dipped into the sample, which allows the sample to be loaded into the microchannels via capillary action. Thirdly, the device is left in incubation to allow the reaction between the analytes of interest and the reagents to occur and produce a colourimetric signal. Finally, the device is placed under a smartphone, and a photo is captured, which is further analyzed via image processing. The simplicity of the chip-and-dip operation procedure makes the device potentially useful for clinicians in point-of-care settings.

## 3. Results and Discussion

### 3.1. Characterization of the PVA Coating in Microchannels

**Figure 2:**
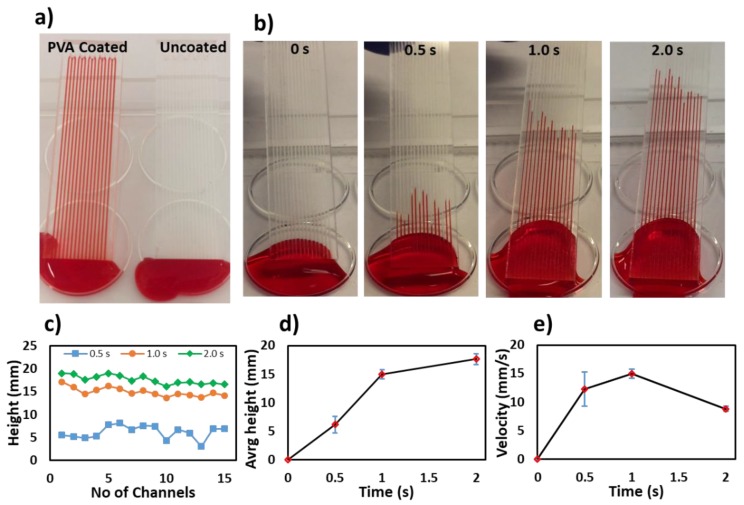
Characterization of the polyvinyl alcohol (PVA) coating and fluid rise in the microchannels. (**a**) Photograph of the uncoated and PVA-coated microchannels. PVA coating renders the polymethyl methacrylate (PMMA) surface hydrophilic and allows a liquid to rise in the microchannels. (**b**) Time-lapse photos of a red food dye rising in PVA-coated microchannels at different times (0, 0.5, 1.0 and 2.0 s). (**c**) Height of the 15 microchannels in a single chip at multiple times (0.5, 1.0 and 2.0 s). (**d**) Graph of time versus average fluid height in the 15 microchannels, showing an increase in height with time. (**e**) Graph of time versus filling velocity in the 15 microchannels.

To test the surface hydrophilicity of PVA-coated microchannels, coated and uncoated microchannels were dipped into a red food dye (East End Foods plc, UK) to observe the liquid rise in the microchannels. The liquid rose only in PVA-coated microchannels, as shown in Figure 2a. Snapshots of the liquid rising in 15 microchannels were taken at multiple times (0, 0.5, 1.0 and 2.0 s), and the speed of the liquid rising in each microchannel was measured. Figure 2b shows the speed of the liquid rising in the microchannels (12 mm/s), with a variability in the 15 microchannels of less than 5% (%RSD). The microchannels were filled within 4 s (4.5 cm-long), as shown in time-lapse photos in Figure 2b. The variation in height and velocity was the highest at 0.5 s, which can be attributed to the slow and abrupt loading of the microchannels at the start of the dipping experiment. The velocity initially increased up to 1.0 s and then decreased. As the microchannels filled, the resistance to the liquid movement increased, and the capillary flow decreased [1]. The variations in the speed measurements can be attributed to differences in the PVA coating, position of the microchannels in the liquid (difference in fluid pressure at the inlet) and reagents cross-linking inside the microchannels.

### 3.2. Characterization of Reagent Cross-Linking inside the Microchannels

**Figure 3:**
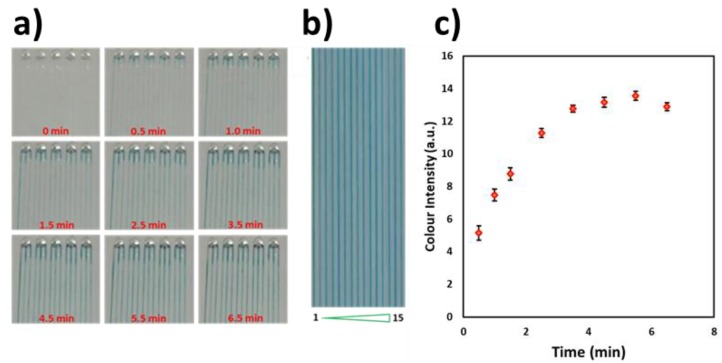
Horseradish peroxidase (HRP)-cortisol-coated microchip dipped in a 3,3′,5,5′-tetramethylbenzidine (TMB) solution. (**a**) Time-lapse photos of the cortisol assay progressing in the microchannels at different times (0.5, 1.0, 1.5, 2.5, 3.5, 4.5, 5.5 and 6.5 min), (**b**) Still photograph after completion of the reaction at 6.5 min. Colour intensity variability in the 15 channels was found to be 2% (%RSD). (**c**) Graph showing the colour intensity change at different times.

A solution of TMB (ThermoScientific, 1 Step Ultra TMB ELISA) was loaded into the microchannels (coated with PVA and cross-linked with glutaraldehyde as previously described) and left for 30 min at room temperature. The microchip was then dipped into a horseradish peroxidase-cortisol (HRP-cortisol) solution (United immunoassay, Cortisol Conjugate (HRP)), and the blue colour was observed (Figure 3a,b). The reagents were used without any further dilution. HPR conjugated to cortisol catalyzes the conversion of TMB, a chromogenic substrate, into a blue-green product. The blue colour intensity increased with time, as shown in Figure 3a, and the colour intensity levels variability was found to be 2% (%RSD) between the 15 microchannels and 7% (%RSD) between the microchips (*n* = 3). Figure 3b shows the colour intensity change at different times (0.5, 1.0, 1.5, 2.5, 3.5, 4.5, 5.5 and 6.5 min) for the 15 microchannels in a single microchip.

**Figure 4:**
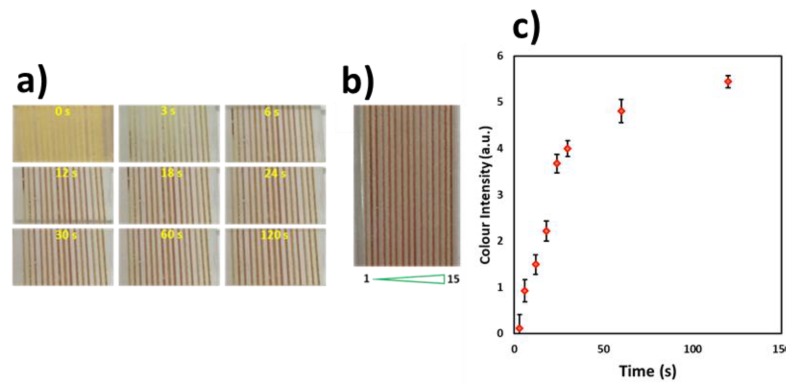
Nitrocefin-coated microchip dipped in a β-lactamase solution. (**a**) Time-lapse photos of the reaction at different times (3, 6, 12, 18, 24, 30, 60 and 120 s), (**b**) Still photograph after completion of the reaction at 2 min. (**c**) Graph showing the colour intensity change at different times. The colour intensity variability in the 15 channels was found to be 5% (%RSD).

Furthermore, the presence of β-lactamase, an indicator of antimicrobial resistance [23,24] was tested by dipping nitrocefin-coated microchannels into a β-lactamase solution (Abcam, recombinant *Escherichia coli* β-lactamase protein) and DI water samples. Nitrocefin (0.25 mg/mL in dimethyl sulfoxide) was loaded into the microchannels and left for 30 min at room temperature. The microchip was then dipped into a solution of β-lactamase (0.2 mg/mL) prepared in phosphate-buffered saline (PBS) at a pH of 7.2. Nitrocefin converted β-lactamase into a red product within 2 min, while the water samples remained yellow (Figure 4a,b). The red colour intensity increased with time (Figure 4a), and the colour intensity levels variability was found to be 5% (%RSD) between the 15 microchannels and 9% (%RSD) between the microchips (*n* = 3). Figure 4c shows the graph of colour intensity change at different times (3, 6, 12, 18, 24, 30, 60 and 120 s) for the 15 microchannels in a single microchip.

A smartphone (iPhone XR) was used to capture images of the microchip under external light, which could add variations in the light signal of the different microchannels. Smartphones can have a variety of optical components, and the quality of an image can be different, which can also add variability to the colour measurements. These variations can be reduced by placing the microchips in a light-controlled box with even light or a white background in a black box and running multiple controls/standards in a single device to generate a calibration curve [21]. In this study, we only focused on the reproducibility of the reaction in between microchannels, and the colour was read by image analysis (ImageJ, NIH) while considering the area between the microchannels as background [25]. However, negative controls and multiple concentrations of analytes should be loaded in the microchannels for a sensitive and quantitative detection of analytes, which we plan to report in future publications.

The chip-and-dip device can be fabricated with multiple sample-loading options and/or with microchannels coated with multiple reagents to capture analytes from a single sample. The mass production of chip-and-dip devices could allow for cheaper analyses at the point-of-care and can be combined with smartphones or portable detectors to provide results near the patients. These microchips can also be coated with reagents and stored away for the detection of various analytes in the clinical settings, such as the determination of minimum inhibitory concentrations (MIC) in microbial assays or antimicrobial susceptibility testing (AST). Our chip-and-dip platform technology is ideally suited to analyse bodily fluids such as urine, tissue dialysate, milk or fisheries water but not blood. This is mainly due to the colorimetric assays used for β-lactamase detection in the microchannels. However, it can be used to analyse clinical blood samples if it is combined with membranes that allow the collection of only serum into the microchannels. This will be further explored in our future studies.

## 4. Conclusions

The chip-and-dip device offers a simple and pump-free method to collect samples into microchannels, making it possible to perform antimicrobial resistance (AMR) testing at the point-of-care. The simple-to-operate nature of this device, its robustness and ease of operational procedures, combined with the portable/handheld detectors which facilitate the analyses can lead towards the development of point-of-care diagnostics for AMR testing in healthcare. The chip-and-dip device design, fabrication and working principle have been illustrated along with specific applications consisting of cortisol and β-lactamase assays. The chip-and-dip device works on the principle of capillary-driven flow microfluidics and allows detection by multiple microchannels in a single microchip via smartphone imaging/portable detectors. Compared to other types of devices such as dipsticks and paper microfluidic devices, our device is fabricated with cheaper materials, coated with minute amounts of reagents and offers multiplexity on a single microchip. The sample is loaded into the microchannels via capillary force, which eliminates the requirement of external/internal fluidic mechanisms or controls. The current device has the potential to be used for detecting β-lactamase-based antimicrobial resistance. Moreover, the multiplexing capability of the device can allow the quantification of specific analytes in a single microchip, which is currently being explored in our lab.
